# Separate and combined analysis of successive dependent outcomes after breast-conservation surgery: recurrence, metastases, second cancer and death

**DOI:** 10.1186/1471-2407-10-697

**Published:** 2010-12-31

**Authors:** Virginie Rondeau, Simone Mathoulin-Pélissier, Lucie Tanneau, Annie J Sasco, Gaétan MacGrogan, Marc Debled

**Affiliations:** 1 INSERM, U897 (Biostatistique), ISPED, Bordeaux, F-33076, France; 2 Université Victor Segalen Bordeaux 2, Bordeaux, F-33076, France; 3 Institut Bergonié - Centre Régional de Lutte Contre le Cancer du Sud-Ouest, Bordeaux, F-33076, France; 4 INSERM, U897 (Team of Epidemiology for Cancer Prevention), Bordeaux, F-33076, France

## Abstract

**Background:**

In the setting of recurrent events, research studies commonly count only the first occurrence of an outcome in a subject. However this approach does not correctly reflect the natural history of the disease. The objective is to jointly identify prognostic factors associated with locoregional recurrences (LRR), contralateral breast cancer, distant metastases (DM), other primary cancer than breast and breast cancer death and to evaluate the correlation between these events.

**Methods:**

Patients (n = 919) with a primary invasive breast cancer and treated in a cancer center in South-Western France with breast-conserving surgery from 1990 to 1994 and followed up to January 2006 were included. Several types of non-independent events could be observed for the same patient: a LRR, a contralateral breast cancer, DM, other primary cancer than breast and breast cancer death. Data were analyzed separately and together using a random-effects survival model.

**Results:**

LRR represent the most frequent type of first failure (14.6%). The risk of any event is higher for young women (less than 40 years old) and in the first 10 years of follow-up after the surgery. In the combined analysis histological tumor size, grade, number of positive nodes, progesterone receptor status and treatment combination are prognostic factors of any event. The results show a significant dependence between these events with a successively increasing risk of a new event after the first and second event. The risk of developing a new failure is greatly increased (RR = 4.25; 95%CI: 2.51-7.21) after developing a LRR, but also after developing DM (RR = 3.94; 95%CI: 2.23-6.96) as compared to patients who did not develop a first event.

**Conclusion:**

We illustrated that the random effects survival model is a more satisfactory method to evaluate the natural history of a disease with multiple type of events.

## Background

Several randomized trials have shown that breast-conserving surgery (BCS) plus locoregional radiotherapy is an appropriate method of primary therapy for the majority of stage I and II breast cancers and may be preferable to radical mastectomy because it provides survival equivalence while conserving the breast [1]. The main concern for both physicians and patients is therefore the risk of local or metastatic recurrence in the conserved breast or in the contralateral breast.

The risk of recurrence (local, locoregional or contralateral), distant metastases (DM) or second primary cancer after breast cancer and their predicting factors has already been documented in the literature [2-6]. In most applications, Cox proportional hazard models are fitted and prognostic factors are studied for each specific event [3]. The most straightforward approach in these recurrent event settings is simply to count only the first occurrence of an outcome in a subject [5]. This analytical strategy is straightforward, and it avoids the methodological complications that can occur if a first event affects either the risk of subsequent events or compliance with ongoing treatment [7]. However, consideration of only first events is not satisfactory to evaluate the natural history of a disease and the effects of therapeutic interventions, furthermore, this is inefficient because it does not utilize all the available information. The potential benefits in terms of events prevented by a treatment can be underestimated if only the first failure is considered.

The solution could be to include as time-dependent covariates the different intermediate events which may affect the patient's event of interest and to study their prognostic impact [4,8]. Yet, interpretation of these time-dependent covariates can be difficult in practice. In this setting of semi-competing risk events, Cox proportional hazard models are simply used for each of the events [9], yet they are unable to estimate the association between these multiple events.

This article hypothesizes that Cox proportional hazards models are not the most appropriate for studying the evolution of breast cancer-related events occurring in a patient. For a more detailed interpretation, one should consider all events. The latter are dependent, for example ipsilateral breast tumor recurrence may be associated with subsequent DM and worse survival [4,10]. A frailty model is proposed to identify the prognostic factors of breast cancer recurrences, metastases and second primary cancer among women treated by BCS for a primary invasive breast cancer. Multiple events in the same subject are considered so it is important to control for the correlation of recurrent events of different types. The frailty model is suitable for studying these recurrent events and prevents biased inferences [11]. In cancer studies, population heterogeneity can be particularly important because of different exposures to carcinogens, different genetic predispositions [12] and the considerably varying speed with which the disease evolves. This population variability will be expressed by some women remaining free of recurrences throughout follow-up and by others having frequent events. Such frailty models could identify the risk of metastases and the need for an adjuvant systemic treatment after a local recurrence. By examining the association between various breast cancer-related events, it may be possible to develop preventive interventions. Furthermore, the statistical power is increased when all available outcomes are considered together instead of using separate statistical analyses for each subsequent outcome.

## Methods

### Patient characteristics and events

All female patients with a primary invasive breast cancer and treated with BCS in a regional comprehensive cancer center (South-West France) from 1990 to 1994 and followed up to January 2006 (n = 1015) were prospectively included in a clinical and pathological database [13,14]. Follow-up included a routine quarterly visit for 2 years, every 6 months for the next 4 years and annually thereafter [13]. Other visits could take place if necessary. If present, a recurrence could be detected at each visit. Patients with bilateral disease (n = 25, left and right breasts with cancer) and patients with past history of breast cancer (n = 71) were excluded from the study (no data on the first tumor were available). Final analyses were based on 919 women.

Up to five non-independent events could be observed for the same patient and were of interest: a locoregional recurrence (LRR), a contralateral cancer (in the opposite breast), distant metastases, other primary cancer than breast, and breast cancer death. An LRR was defined as any recurrence of cancer in the ipsilateral breast or chest wall or regional lymph nodes including the axillary, supra/infraclavicular and internal mammary nodes.

The analyses were adjusted non-parametrically for age, ie., age was chosen as the basic timescale. This allows to take into account an important risk factor of recurrence, without making parametric assumptions about the effect of this variable. A subject was considered at risk for the k^th ^failure from her age at the k-1^th ^failure to her age at the k^th ^failure or her age of censorship. The following prognostic factors were analyzed in the multivariate context: lymphadenectomy (yes/no); initial treatment combination; clinical tumor size; histological tumor size; tumor stage (TNM classification); tumor grade (in 3 categories), histological type ((1) infiltrating ductal, (2) infiltrating ductal with extensive intraductal component (> 80%), (3) infiltrating lobular (including mixed types), and (4) other types (i.e., medullary, colloid, and tubular carcinomas); surgical excision margin status; node involvement; estrogen receptor status; progesterone receptor status; lympho-vascular invasion. Demographic features were collected and analyzed: family history of cancer or breast disease, parity.

Ethical approval from the national ethics committee (Commission Nationale de l'Informatique et des Libertés) was obtained for this study, which allowed the use of data recorded in this clinical and pathological database. In this comprehensive cancer center, each patient was informed that medical data can be use in observational research. The procedure follows the French law for medical research.

### Statistical methods

In the first analysis the five outcomes of interest were analyzed separately using a Cox proportional hazard model [15]. Age was taken as the basic time scale in the analysis, so the risk of recurrence was adjusted non-parametrically for age. This has two implications: firstly, it enables inferences on the effects of prognostic factors to be made without making parametric assumptions about the effects of age and secondly, the hazard functions are the age-specific incidences. A subject was considered at risk from her age at surgery to her age at censorship or age at outcome. A Cox proportional hazard model with delayed entry (left truncation) was performed to estimate relative risks (RR) and to adjust for covariates [16]. It considers that women are at risk for an observable recurrence only after their age of surgery, and not from birth. The recurrence hazards functions will be represented according to age or to the time since surgery. Patients with no failure and lost to follow-up, alive at the study termination date (May 31^st ^2006) or who had died were censored at their last known follow-up time for either event of interest.

In a second analysis, all outcome types by subject were analyzed together using a random-effects survival model [17]. This type of model incorporates the two features of recurrent event processes: heterogeneity among individuals and event dependence. This produces correlations between recurrences. Heterogeneity is produced because some subjects have a higher (or lower) event rate than other subjects due to unknown or nonmeasurable effects. The frailty term (its variance) takes into account these genetic, hormonal or environmental factors which can lead to a positive association between our five events of interest. Further, the occurrence of a given event may make further relapses more or less likely. This event dependence may be acquired or defined by biological characteristics and may be modeled using time-dependent covariates. We adjusted for the number of prior events as categorized variables (3 binary variables for 4 classes). This frailty model also better reflects the true clinical course of the disease in this heterogeneous population (see the "Statistical Appendix" section). As a result, analyses that fail to account for the correlation between survival times for each subject are likely to underestimate the variances of the parameters.

In all analyses, tests were two-sided and evidence was considered to be statistically significant at the 5% level. Individual characteristics were selected based on descending stepwise multiple regressions. Analyses were conducted using SAS software, version 9.1 (SAS Institute, INC., Cary, North Carolina). The frailty models were implemented with the R package "frailty pack" (publicly available and free to download at http://cran.r-project.org).

## Results

Patient characteristics are described in Table 1. Median age was 57 years (29 years to 87 years, mean 56.7). Axillary node dissection has been performed in 882 cases (96.0%). Surgery was followed by post-operative breast irradiation in 913 cases (99.4%). Adjuvant therapy with chemotherapy and/or endocrine treatments were prescribed according to the menopausal status of the patient, nodal status and hormone receptor results.

**Table 1:** Patient characteristics and event distribution in relation to patient, clinical, tumor and treatment factors

Characteristic	No. of patients
Age, y	
[29-39]	52 (5.7%)
[40-49]	227(24.7%)
[50-69]	512(55.7%)
≥ 70	128(13.9%)
Total	919
Histological size of the tumor (mm)	
25^th ^quartile	11.0
Median	15.0
75^th ^quartile	20.0
Range	1-45
Total	881
Extensive intraductal component (EIC)	
-No	866 (94.2%)
-Yes	53 (5.8%)
Total	919
Grade	
- Grade 1	324 (40.0%)
- Grade 2	355 (43.8%)
- Grade 3	131 (16.2%)
Total	810
# positive nodes	
- 0	549 (62.8%)
- 1-3	247 (28.3%)
- ≥4	78 (8.9%)
Total	874
Lympho-vascular invasion	
- No	667 (75.8%)
- Yes	213 (24.2%)
Total	880
Surgical excision margin status	
- Clear (ref)	549 (63.7%)
- Invasion	313 (36.3%)
Total	862
Progesterone receptor status	
- Positive	459 (55.0%)
- Negative	376 (45.0%)
Total	835
Estrogen receptor status	
- Positive	657 (21.5%)
- Negative	180 (78.5%)
Total	837
Treatment combination	
- Radiotherapy	586 (63.8%)
- Radio- and chemotherapy	129 (14.1%)
- Radio- and hormonal therapy	183 (19.9%)
- Radio- and chemo- and hormonal therapy	20 (2.2%)
Total	918

Median follow-up was 12.7 years (150 days to 16 years, mean: 11.8 years). Over the five non-independent events studied, we observed 150 locoregional recurrences, 69 contralateral cancers, 188 distant metastases, 30 other primary cancers than breast, and 133 breast cancer deaths. During the follow-up 580 (63.1%) patients did not experience any event of interest (Table 2). At the end of the study, 209 patients (22.7%) had died and 2 (0.2%) were lost to follow-up. The last line in Table 2 shows that the duration between two successive events decreased with follow-up time. The median age at entrance (53.0 years vs. 71.5 years) was lower for those who had died from their breast cancer (n = 133) compared to those who had died from other causes (n = 76).

**Table 2:** Mean follow-up times in years between two successive events (gap times) according to the type of event: locoregional, contralateral breast cancer, metastases, 2nd cancer other than breast or breast cancer death

Event of interest	Entry-first event		1^st ^- 2^nd ^event		2nd - 3rd event		3rd - 4th event		ANY EVENT after surgery	
	Gap time* (SD)	**N** **(%)**	**Gap time** **(SD)**	**N** **(%)**	**Gap time** **(SD)**	**N** **(%)**	**Gap time** **(SD)**	**N** **(%)**	**Gap time** **(SD)**	**N** **(%)**

Locoregional	5.85 (3.60)	134 (14.6%)	2.57 (2.13)	15 (4.4%)	7.52 -	1 (1.1%)	- -	0 (0%)	5.53 (3.60)	150 (11.1%)
Contralateral breast cancer	5.60 (3.45)	63 (6.9%)	1.39 (1.54)	6 (1.8%)	- -	0 (0%)	- -	0 (0%)	5.23 (3.53)	69 (5.1%)
Metastases	5.23 (3.29)	117 (12.7%)	1.14 (1.68)	64 (18.9%)	1.10 (1.48)	7 (7.9%)	- -	0 (0%)	3.68 (3.42)	188 (13.9%)
2^nd ^cancer other than breast	4.71 (2.64)	25 (2.7%)	0.75 (0.71)	3 (0.9%)	1.71 (2.42)	2 (2.3%)	- -	0 (0%)	4.12 (2.80)	30 (2.2%)
Death from cancer	1.56 (.)	1 (0.1%)	2.14 (1.81)	76 (22.4%)	1.83 (1.62)	52 (59.1%)	1.21 (1.97)	4 (40%)	1.99 (1.71)	133 (9.8%)
Censoring**	10.83 (2.31)	580 (63.1%)	3.41 (3.08)	251 (74.0%)	2.38 (2.20)	78 (88.6%)	1.47 (1.37)	10 (100%)	7.99 (4.51)	919 (67.8%)
**TOTAL**	**8.87** **(3.78)**	**919** **(100%)**	**2.89** **(2.94)**	**339** **(100%)**	**2.32** **(2.22)**	**88** **(100%)**	**1.47** **(1.37)**	**10** **(100%)**	**6.89** **(4.51)**	**1356** **(100%)**

* Gap time = time between two events

** censoring by death from other causes or lost to follow-up or alive at study termination

The number of events ranged from 0 to 4 (average = 0.62) per patient. The first failure type was mostly LRR (n = 134, 14.6%). Second failures were most commonly death from breast cancer (n = 76, 22.4%) for all first failure types. The most frequent event observed over the whole follow-up was metastases, which was observed in 188 of the women (13.9% of the total number of events observed).

### Analysis of each event separately

The results of the five separate analyses for each event of interest are shown in Table 3.

**Table 3:** Results of the 5 separate Cox regression analyses to evaluate the prognostic factors associated with the risk of locoregional recurrence, contralateral breast cancer, metastases or 2nd cancer other than breast

Characteristic	Locoregional recurrences		Contralateral breast cancer		Distant metastases		2^nd ^primary cancer other than breast		Breast cancer death	
	**RR** **(95% CI)**	**p**	**RR** **(95% CI)**	**P**	**RR** **(95% CI)**	**P**	**RR** **(95% CI)**	**p**	**RR** **(95% CI)**	**p**

Histological size of the tumor (for 1 mm increase)	1.02 (0.99-1.06)	0.17	1.02 (0.97-1.06)	0.54	1.02 (1.00-1.05)	0.08	1.01 (0.93-1.10)	0.75	1.02 (0.99-1.05)	0.27
Extensive intraductal component (EIC)										
- No (ref)	1		1		1		--		1	
- Yes	3.34 (0.89-12.5)	0.07	0.99 (0.13-7.46)	1.00	1.55 (0.34-7.06)	0.57	--	--	1.43 (0.20-10.8)	0.73
Grade										
- Grade1(ref)	**1**		1		1		1		**1**	
- Grade 2	**1.84** **(1.11-3.07)**	**0.02**	1.45 (0.78-2.70)	0.24	**2.68** **(1.58-4.53)**	**< 0.001**	0.68 (0.23-2.01)	0.48	**3.64** **(1.74-7.60)**	**< 0.001**
- Grade 3	**2.12** **(1.17-3.84)**	**0.01**	0.94 (0.40-2.21)	0.88	**3.39** **(1.91-6.02)**	**< 0.001**	1.32 (0.37-4.75)	0.67	**5.54** **(2.58-11.9)**	**< 0.001**
# positive nodes										
- 0 (ref)	1		1		**1**		--		**1**	
- 1-3	**2.60** **(1.30-5.19)**	**0.007**	1.17 (0.34-3.97)	0.81	**2.41** **(1.29-4.47)**	**0.006**	--	--	**2.92** **(1.50-5.68)**	**0.002**
- ≥4	**4.40** **(1.80-10.7)**	**0.001**	0.66 (0.13-3.45)	0.62	**6.91** **(3.41-14.0)**	**< 0.001**	--	--	**8.03** **(3.71-17.4)**	**< 0.001**
Lympho-vascular invasion										
- No (ref)	1		**1**		**1**		1		1	
- Yes	1.29 (0.84-1.98)	0.24	**0.41** **(0.19-0.90)**	**0.03**	**1.47** **(1.04-2.08)**	**0.03**	1.40 (0.51-3.83)	0.51	1.27 (0.87-1.91)	0.26
Surgical excision margin status										
- Clear (ref)	1		**1**		1		1		1	
- Invasion	0.95 (0.64-1.43)	0.81	**2.36** **(1.40-3.98)**	**0.001**	1.05 (0.74-1.48)	0.78	0.57 (0.19-1.73)	0.32	1.12 (0.75-1.67)	0.59
Progesterone receptor status										
-Positive(ref)	1		1		1		1		**1**	
- Negative	1.26 (0.84-1.88)	0.26	1.21 (0.70-2.09)	0.49	1.32 (0.93-1.86)	0.12	0.33 (0.11-1.03)	0.06	**1.90** **(1.26-2.87)**	**0.002**
Treatment combination										
- Radiotherapy (ref)	**1**		1		1		--		1	
- Radio- and chemotherapy	**0.23** **(0.11-0.50)**	**< 0.001**	1.22 (0.33-4.55)	0.77	0.52 (0.27-1.02)	0.06	--	--	0.56 (0.27-1.16)	0.12
- Radio- and hormonal therapy	**0.35** **(0.16-0.77)**	**0.009**	0.78 (0.21-2.91)	0.71	0.59 (0.30-1.13)	0.11	--	--	**0.48** **(0.24-0.99)**	**0.05**
- Radio- and chemo- and hormonal therapy	**0.06** **(0.01-0.49)**	**0.008**	0.74 (0.07-7.60)	0.80	**0.33** **(0.11-0.99)**	**0.05**	--	--	0.28 (0.07-1.06)	0.06

-- variables not included in the model

They suggest that the risk of LRR was higher for women with higher tumor grade, with an extensive intraductal component or with nodal invasion. In patients treated with a combination of radiotherapy, chemotherapy and hormonotherapy, less locoregional occurrences were observed.

The risk of contralateral breast cancer was increased in patients showing involvement of excision margins after BCS (RR = 2.36, 95%CI = 1.40-3.98). By contrast, lympho-vascular invasion was inversely associated with contralateral breast cancer.

With regard to factors influencing distant metastases, it was found that grade, nodal invasion and lymphovascular invasion were highly prognostic factors of distant metastases. In patients treated with a combination of radiotherapy, chemotherapy and hormonotherapy less metastases were observed.

No predictive factors except the progesterone receptor status were associated with the risk of developing a new second primary cancer other than breast carcinoma, but a poor statistical power may explain these results (only 30 new second primary cancers were observed).

Grade and number of positive nodes were also associated with breast cancer death. Progesterone receptor positivity decreased the risk of death from breast cancer.

The following factors were not associated with any of the five events of interest and were not retained in analyses in Table 3 and 4: histological type, family history of cancer or breast disease, parity, estrogen receptor status.

**Table 4:** Results of the frailty models to evaluate jointly the prognostic factors associated with any of the 5 types of failures: locoregional recurrence, contralateral breast cancer, metastases, 2nd cancer other than breast or breast cancer death

	Model 1	Model 2	Model 3
Characteristic*	**RR** **(95% CI)**	**RR** **(95% CI)**	**RR** **(95% CI)**

Histological size of the tumor (for 1 mm increase)	**1.03** **(1.01-1.06)**	**1.02** **(1.00-1.04)**	**1.02** **(1.00-1.04)**
Extensive intraductal component (EIC)			
- No (ref)	1	1	1
- Yes	1.91 (0.58-6.24)	1.44 (0.58-3.59)	1.21 (0.49-3.01)
Grade			
- Grade 1 (ref)	**1**	**1**	**1**
- Grade 2	**2.06** **(1.44-2.95)**	**1.77** **(1.30-2.40)**	**1.69** **(1.24-2.29)**
- Grade 3	**3.19** **(2.08-4.87)**	**2.41** **(1.69-3.43)**	**2.32** **(1.64-3.29)**
# positive nodes			
- 0 (ref)	**1**	**1**	**1**
- 1-3	**2.82** **(1.62-4.91)**	**2.33** **(1.52-3.57)**	**2.33** **(1.52-3.55)**
- ≥4	**6.16** **(3.15-12.04)**	**4.60** **(2.74-7.72)**	**3.97** **(2.36-6.66)**
Lympho-vascular invasion			
- No (ref)	1	1	1
- Yes	1.10 (0.80-1.52)	1.08 (0.85-1.38)	1.02 (0.80-1.30)
Surgical excision margin status			
- Clear (ref)	**1**	1	1
- Invasion	**1.36** **(1.02-1.82)**	1.21 (0.96-1.52)	1.23 (0.97-1.54)
Progesterone receptor status			
- Yes (ref)	**1**	**1**	**1**
- No	**1.45** **(1.09-1.93)**	**1.43** **(1.14-1.80)**	**1.46** **(1.16-1.83)**
Treatment combination			
- Radiotherapy (ref)	**1**	**1**	**1**
- Radio- and chemotherapy	**0.50** **(0.27-0.91)**	**0.55** **(0.35-0.87)**	**0.59** **(0.37-0.93)**
- Radio- and hormonal therapy	**0.36** **(0.19-0.66)**	**0.50** **(0.31-0.81)**	**0.54** **(0.34-0.86)**
- Radio- and chemo- and hormonal therapy	**0.22** **(0.08-0.63)**	**0.29** **(0.12-0.67)**	**0.31** **(0.13-0.72)**
Number of prior events (as a quantitative variable)	-	**1.97** **(1.62-2.41)**	0.96 (0.67-1.41)
Type of prior events:			
- no previous event (ref)	-	-	**1**
- locoregional	-	-	**4.25** **(2.51-7.21)**
- contralateral breast cancer	-	-	1.44 (0.75-2.74)
- distant metastases	-	-	**3.94** **(2.23-6.96)**
- 2^nd ^primary other than breast			- (-)
Variance of the random effect	**1.42 (0.24)**	**0.38 (0.20)**	**0.35 (0.17)**

Akaike Information Criterium (AIC)	*1518.98*	*1494.10*	*1461.69*

* Analyses parametrically adjusted for the characteristics mentioned and non-parametrically adjusted for age

**Figure 1:**
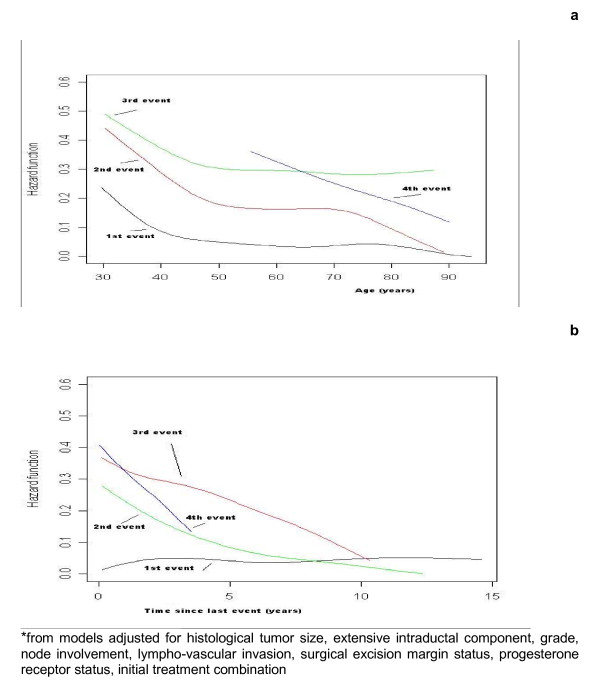
Age-specific* (a) and time-specific* (b) recurrence hazards after a breast cancer treated by breast-conserving surgery for the first, second, third or fourth event.

**Figure 2:**
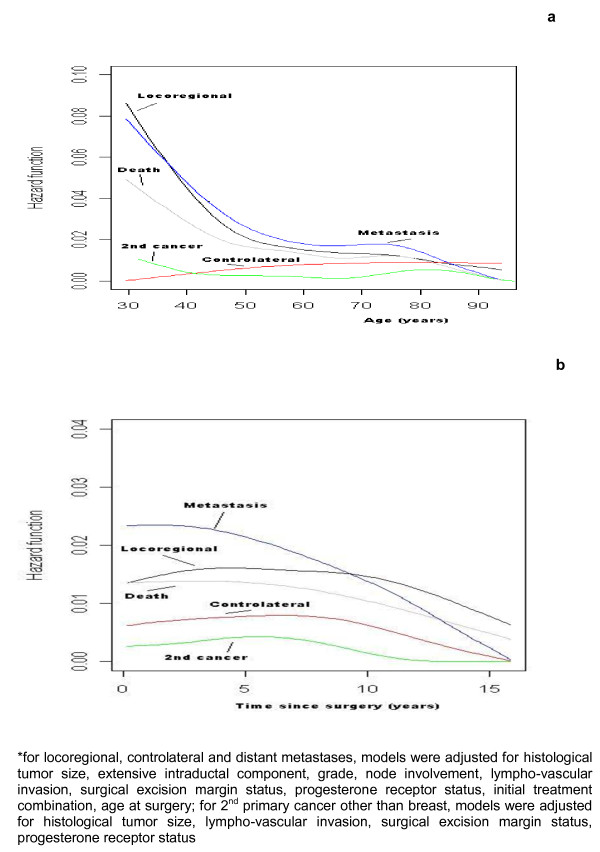
Age-specific* (a) and time-specific* (b) recurrence hazards after a breast cancer treated by breast-conserving surgery according to the type of relapse.

Figures 1 and 2 show the baseline recurrence hazards generated from adjusted models, according to the rank of the recurrence or the type of the recurrence. Figure 1a shows that the first two baseline age-specific recurrence hazards are higher for young women (less than 40 years old). It also shows that at any age the risk of failure (recurrence, contralateral breast cancer, metastases, 2nd primary other than breast or death) increased successively with the number of prior events. For instance, the risk of developing a third event if a patient had already developed a second event was higher than the risk of developing a second event if she had already developed a first event. Figure 1b illustrates the four separate time-specific recurrence hazards and shows that the risk of developing a first relapse event is fairly stable across time since surgery. The time to first event is longer than the other gap times (i.e. time between two successive events) and can therefore occur at an older age.

Figures 2a and 2b show the 5 type-specific hazards (for recurrence, contralateral breast cancer, metastases, 2^nd ^cancer other than breast or death from breast cancer) without taking into account the rank of failure. The different types of events follow different patterns across the time intervals. The risks of developing LRR or DM is higher than the risks of developing other events at any time and at any age. A greater risk of metastases was observed during the first 10 years after surgery (see Figure 2b) but the other types of failure occurred at a constant rate over at least 10 years. After that time, the risk of failure decreases progressively.

### Combined analysis of all events

We analyzed the successive failures for each subject and their dependence using a random effects analysis (Table 4, with or without adjustment for the number of prior events or the type of failure). The rate of failure increased with grade (grade 2 vs. grade 1, RR = 1.77, 95% CI = 1.30-2.41; grade 3 vs. grade 1, RR = 2.41, 95% CI = 1.69-3.43 in model 2) and was higher for women with nodal invasion and with radiotherapy alone. The histological tumor size was associated with the rate of failure; this association was not previously observed in the 4 separate analyses (see Table 3). We observed a significant association between the treatment intervention and the risk of developing an event of interest, with decreasing risks when chemotherapy and hormonal therapy were added to locoregional treatment.

The number of previous events (as a quantitative variable) was significantly associated with an increased risk of failure given the frailty term, but was no longer significant after adjustment for the type of failure. The coding of the number of prior events as categorical variables confirmed the above mentioned relationships. The tendency was towards a higher risk of second failure compared to the risk of first failure.

Heterogeneity of the survival times was observed in model 1 (Table 4, see variance of the random effects) after adjustment for the individual variables. The variance of the random effects (0.38) decreased in model 2 after adjustment for the prior number of events but remained significantly different from zero. Model 3 (Table 4) shows the influence of the type of first event: the risk of developing a new failure is greatly increased (RR = 4.25) after developing a LRR, but also after developing DM (RR = 3.94) as compared to patients who did not develop a first event. The small number of patients with a second primary cancer other than breast and followed by an event (n = 1) makes this variable estimate unreliable.

## Discussion

This study uses a novel approach of analyzing together the different rates of failure following successive events for women with breast cancer treated with BCS. The risk of relapse of any type was higher for young women (less than 40 years old) and is higher in the first 10 years after surgery. Traditionally guidelines for follow-up of breast cancer patients concentrate on the first 3-5 years, with either reduced frequency of visits or discharge after this. Montgomery et al. [18] show that the basic assumptions behind these generally-accepted guidelines for follow-up may be incorrect. They suggest that if the central aim is the detection of treatable relapse, as stated in the guidelines, then there is no justification for focusing on the first 2-3 years after initial therapy, as treatable relapses occur at a constant rate over at least 10 years. Our results are in accordance with these results and the implications for cancer care should be carefully considered.

As this was a prospective observational study in a single center, there are potential limitations to our results. Principal among these limitations could be a bias for patient selection or for their follow-up. In terms of patient selection, we noted a median age (57 years) lower than the median age incidence in France [19] (61 years), but we selected only invasive breast cancer. The potential bias for follow up in these types of studies appears not to be the case in this study as the cancer center employs a rigourous clinical follow-up procedure and we administered a survey to physicians resulting in less than 2% losses to follow-up [13].

This frailty model allows the analyst to estimate simultaneously and distinctly the effects of both unobserved heterogeneity through a subject-specific random effect and event dependence through a time-dependent covariate. It also helps us to diagnose the source of correlation in the data by comparing the magnitude of the estimated effects across the models, i.e. the adjustment to some covariates will completely explain the unobserved heterogeneity. In this typical repeated events context, the usual Cox model was both biased and inefficient and the five separate analyses were uninformative with regard to heterogeneity across individuals and event dependence. Furthermore, the combined analysis increased the statistical power to such an extent that the associations of some prognostic factors with any event were revealed (ex: histological tumor size in Table 4). No separate analysis was able to do this. Since the risk of any recurrence was higher for young women and in the first 10 years after surgery, this justifies the need for increased surveillance of women in the first years following the diagnosis of cancer. Tai et al. [20] recently proposed a marginal approach in order to utilize fully all the available information on event times and not only the information on the first event that occurs. But, their approach does not measure the dependence among the multiple events and the influence of prior events on future failures is not their focus. Recently using a multivariate multistate model, de Bock et al. [21] found that patients with a LRR have a more than threefold increased risk of developing DM as compared to patients who develop no LRR. As with the frailty model, the strongest point of their approach is that all the data are summarized in one model instead of presenting many separate analyses. Presenting the many separate analyses will lower the power of the estimated effects or may result in false positive findings. However such a multistate analysis can only be performed on a large cohort of patients with a long follow-up time (with enough metastatic events after the occurrence of LRR). The frailty model is less restrictive on the number of events.

At the end of the study, 209 patients had died, among them 133 had died from their breast cancer. Deaths could be related to their health status and more specifically to breast cancer recurrences (local or distant). Thus, breast cancer death is likely to be an informative type of censoring, which means that those individuals who are censored by death are not as likely to have the subsequent event of interest as those who remained in the study. In order to examine this hypothesis we used a joint frailty model (using the R package "frailtypack") to analyze recurrent observations of breast cancer with death from breast cancer [22,23]. For instance, a patient recurrence rate may be positively correlated with its death rate. The effects of prognostic factors were unchanged in this joint frailty model. Therefore, censoring by death from breast cancer appears to be non-informative for recurrence after adjustment. This suggests that selection by death from breast cancer in this study did not importantly bias our findings.

In this study we proposed to control for the type of event by adjusting for this variable in the frailty models (see Table 4, model 3). We then observed that patients with a LRR had a more than fourfold increased risk of developing a new event as compared to patients who did not yet develop any event. However in this approach the prognostic factors had the same effects for the different types of event. Another approach could be to use a joint frailty model with one hazard function for each type of event [24]. This would allow us to evaluate the prognostic impact for each type of event and so for each clinical etiology. However we have not considered this extension in the present application, due to the increased computational complexity of the problem.

Using breast cancer data from a population-based cancer registry, Cheung et al. [25] illustrated the features of Cox models using two time scales (time since diagnosis and age). Using time since diagnosis as the time scale, a younger age at diagnosis was associated with a lower mortality, while using age as the time scale gave the opposite result. Age was chosen as the basic time scale in our analysis, so no parametric assumptions were made in the analyses and the hazard function of the time of onset of any recurrence was the age-specific incidence of the recurrence. We also analyzed parametrically the effect of age by introducing age as a covariate in a proportional hazard model (i.e. with a log-linear relationship). The results were quite similar in models adjusted for age with four classes coded by three binary variables (40-49, 50-69, 70 and over versus 29-39 years). Age was associated with the risk of locoregional recurrence or metastases with a higher risk for younger patients. However, if the true effect of age is far from this parametric log-linear assumption, this may lead to spurious results.

Our study seems to show a previously unreported unexplained protective effect of lympho-vascular invasion on the risk of contralateral breast cancer. The epidemiology of contralateral breast cancer is complex to study for a number of reasons. Firstly, the patient has received treatment that may modify the risk of second cancer, and she is under intense medical scrutiny and self-observation [26,27]. Secondly, the treatment and follow-up procedures for breast cancer have changed extensively over the years and several definitions of contralateral breast cancer were observed. This may contribute to the incoherent results reported in the epidemiology of contralateral disease [27,28]. Comparison of genomic profiles would allow differentiation between metastatic lesions and new primary tumor, but this information was not available to us. Microarray studies have shown that distinct molecular profiles can be useful for classifying tumors and predicting outcome [29].

Tailored systemic treatment making use of ER and PR receptors and HER2 is now current practice [30]. However these prognostic factors were missing in our study. Only more recent studies will include the newer predictive and prognostic factors, but these studies will not allow us to perform such long-term analyses due to their shorter follow-up.

## Conclusions

In conclusion, the frailty model approach allows the simultaneous analysis of all successive events intervening after a first breast cancer. This methodology in conjunction with conventional methods represents a valuable tool for describing the natural history of breast cancer and the possible effects of treatment. This methodology issue may also be relevant to studies of other tumor types with recurrences. This approach can also, if data require it, incorporate the effects of interventions which are performed after each event. Response to treatment after each relapse may be an important factor to predict new relapses; this is an important issue which is not considered in the models for recurrent event data.

## Statistical Appendix

We consider models in which the hazard function partly depends on an unobservable random variable thought to act multiplicatively on the hazard, so that a large value of the variable increases the hazard. These models, called frailty models are an extension of the classical Cox proportional hazard model [15].

Suppose there are *N *subjects in a study. For the j^th ^event (j = 1,..., ni) of the i^th ^individual (i = 1,...,N), let *T_ij _*denote the survival times under study and let *C_ij _*be the corresponding right-censoring times. The observations are *Y_ij _*= min(*T_ij_*, *C_ij_*) and the censoring indicator Δ_ij _= *I*(*T_ij _*≤ *C_ij_*) is one if the observation j is a failure and 0 if it is a censoring. Our frailty model specifies that the hazard function conditional on the frailty is:

$$\lambda_{ij}(t|Z_{i},X_{ij})=Z_{i}\lambda_{0}(t)\exp(\beta 'X_{ij})$$

where *λ*_0_(*t*) is the baseline hazard function; *X_ij _*= (*X*_1*ij*_,..., *X_pij_*)' denotes the covariate vector for the j^th ^recurrence of individual i, β is the corresponding vector of regression parameters, and the *Z_i_*'s are unobserved random variables (the frailties). It is assumed for mathematical convenience that the *Z_i_*'s are independently and identically distributed from a gamma distribution with mean 1 and unknown variance θ at time of origin.

Different timescales can be used [31]. The timescale that is most often used is the gap time: after an event, the subject starts again at time 0 and the time to the next event corresponds to the number of days that it takes to experience the next event. An alternative timescale is the calendar time, also called the counting process approach [32] which keeps track of time since randomization. The duration of the time at risk for an event corresponds to the duration of the time at risk in the gap time representation. However, the start of the at-risk period is not reset to 0. A subject is not considered to be at risk for the k^th ^event until after the (k-1)^th ^event. A particular subject has different periods at risk during the total observation time. If there are *n_i _*at-risk periods for patient i, then the complete information for patient i can be represented by *n_i _*triplets $\left(Y_{i11},Y_{i12},\Delta_{i1}),...,(Y_{in_{i}1},Y_{in_{i}2},\Delta_{in_{i}}\right)$ where, for the j^th ^triplet, *Y*_*ij*1 _is the start of the j^th ^at-risk period, *Y*_*ij*2 _is the end of the j^th ^at-risk period, Δ_*ij *_is the censoring indicator and *Y*_*i*11 _= 0. If age is chosen as the basic timescale, the hazard functions estimated are for instance the age-specific recurrence incidences or the age-specific mortality. A subject is considered at risk from her age at surgery (= *Y*_*i*11_) to her age at censorship or failure.

The expression of the full marginal likelihood associated with this frailty model is used. In most situations it is reasonable to expect smooth baseline hazard functions, piecewise constant modeling for the hazard functions often being unrealistic. To introduce such a priori knowledge, we penalize the likelihood. The expression of the full marginal likelihood is developed in expression 4 of Rondeau et al. [23].

## Competing interests

The authors declare that they have no competing interests.

## Authors' contributions

VR provided data analysis, interpretation and manuscript writting

LT provided data analysis

SMP, AJS, GM and MD provided interpretation and manuscript writting

All authors read and approved the final paper.

## Pre-publication history

The pre-publication history for this paper can be accessed here:

http://www.biomedcentral.com/1471-2407/10/697/prepub

## References

[B1] Veronesi U Marubini E Del Vecchio M Manzari A Andreola S Greco M Luini A Merson M Saccozzi R Rilke F Local Recurrences and Distant Metastases after Conservative Breast Cancer Treatments: Partly Independent Events J Natl Cancer Inst 1995 87 19 27 10.1093/jnci/87.1.19 7666458

[B2] Mellemkjaer L Friis S Olsen JH Scelo G Hemminki K Tracey E Andersen A Brewster DH Pukkala E McBride ML Kliewer EV Tonita JM Kee-Seng C Pompe-Kirn V Martos C Jonasson JG Boffetta P Brennan P Risk of Second Cancer among Women with Breast Cancer Int J Cancer 2006 118 2285 2292 10.1002/ijc.21651 16342146

[B3] Komoike Y Akiyama F Iino Y Ikeda T Akashi-Tanaka S Ohsumi S Kusama M Sano M Shin E Suemasu K Sonoo H Taguchi T Nishi T Nishimura R Haga S Mise K Kinoshita T Murakami S Yoshimoto M Tsukuma H Inaji H Ipsilateral Breast Tumor Recurrence (Ibtr) after Breast-Conserving Treatment for Early Breast Cancer: Risk Factors and Impact on Distant Metastases Cancer 2006 106 35 41 10.1002/cncr.21551 16333848

[B4] Touboul E Buffat L Belkacemi Y Lefranc JP Uzan S Lhuillier P Faivre C Huart J Lotz JP Antoine M Pene F Blondon J Izrael V Laugier A Schlienger M Housset M Local Recurrences and Distant Metastases after Breast-Conserving Surgery and Radiation Therapy for Early Breast Cancer Int J Radiat Oncol Biol Phys 1999 43 25 38 10.1016/S0360-3016(98)00365-4 9989511

[B5] de Bock GH van der Hage JA Putter H Bonnema J Bartelink H van de Velde CJ Isolated Loco-Regional Recurrence of Breast Cancer Is More Common in Young Patients and Following Breast Conserving Therapy: Long-Term Results of European Organisation for Research and Treatment of Cancer Studies Eur J Cancer 2006 42 351 356 10.1016/j.ejca.2005.10.006 16314086

[B6] Huston TL Simmons RM Locally Recurrent Breast Cancer after Conservation Therapy Am J Surg 2005 189 229 235 10.1016/j.amjsurg.2004.07.039 15720997

[B7] Glynn RJ Buring JE Counting Recurrent Events in Cancer Research J Natl Cancer Inst 2001 93 488 489 10.1093/jnci/93.7.488 11287433

[B8] Haffty BG Reiss M Beinfield M Fischer D Ward B McKhann C Ipsilateral Breast Tumor Recurrence as a Predictor of Distant Disease: Implications for Systemic Therapy at the Time of Local Relapse J Clin Oncol 1996 14 52 57 8558220 10.1200/JCO.1996.14.1.52

[B9] Broet P de la Rochefordiere A Scholl SM Fourquet A De Rycke Y Pouillart P Mosseri V Asselain B Analyzing Prognostic Factors in Breast Cancer Using a Multistate Model Breast Cancer Res Treat 1999 54 83 89 10.1023/A:1006197524405 10369084

[B10] Meric F Mirza NQ Vlastos G Buchholz TA Kuerer HM Babiera GV Singletary SE Ross MI Ames FC Feig BW Krishnamurthy S Perkins GH McNeese MD Strom EA Valero V Hunt KK Positive Surgical Margins and Ipsilateral Breast Tumor Recurrence Predict Disease-Specific Survival after Breast-Conserving Therapy Cancer 2003 97 926 933 10.1002/cncr.11222 12569592

[B11] Box-Steffensmeier JM De Boef S Repeated Events Survival Models: The Conditional Frailty Model Stat Med 2006 25 3518 3533 10.1002/sim.2434 16345026

[B12] Maillet P Chappuis PO Khoshbeen-Boudal M Sciretta V Sappino AP Twenty-Three Novel Brca1 and Brca2 Sequence Variations Identified in a Cohort of Swiss Breast and Ovarian Cancer Families Cancer Genet Cytogenet 2006 169 62 68 10.1016/j.cancergencyto.2006.03.010 16875939

[B13] de Mascarel I MacGrogan G Mathoulin-Pelissier S Soubeyran I Picot V Coindre JM Breast Ductal Carcinoma in Situ with Microinvasion: A Definition Supported by a Long-Term Study of 1248 Serially Sectioned Ductal Carcinomas Cancer 2002 94 2134 2142 10.1002/cncr.10451 12001109

[B14] de Mascarel I Soubeyran I MacGrogan G Picot V Mathoulin-Pelissier S Immunohistochemically Detected Lymph Node Metastases from Breast Carcinoma: Practical Considerations About the New American Joint Committee on Cancer Classification Cancer 2005 103 1319 1322 10.1002/cncr.20937 15719436

[B15] Cox DR Regression Models and Life Tables (with Discussion) Journal of the Royal Statistical Society: Series B (Statistical Methodology) 1972 34 187 220

[B16] Commenges D Letenneur L Joly P Alioum A Dartigues JF Modelling Age-Specific Risk: Application to Dementia Stat Med 1998 17 1973 1988 10.1002/(SICI)1097-0258(19980915)17:17<1973::AID-SIM892>3.0.CO;2-5 9777690

[B17] Rondeau V Commenges D Joly P Maximum Penalized Likelihood Estimation in a Gamma-Frailty Model Lifetime Data Analysis 2003 9 139 153 10.1023/A:1022978802021 12735493 PMC1961627

[B18] Montgomery DA Krupa K Jack WJ Kerr GR Kunkler IH Thomas J Dixon JM Changing Pattern of the Detection of Locoregional Relapse in Breast Cancer: The Edinburgh Experience Br J Cancer 2007 96 1802 1807 10.1038/sj.bjc.6603815 17533401 PMC2359955

[B19] Trétarre B Guizard AV Fontaine D Francim R CépiDc-Inserm5 Cancer Du Sein Chez La Femme: Incidence Et Mortalité, France 2000 Bulletin épidémiologique hebdomadaire 2004 44 209 210

[B20] Tai BC De Stavola BL de Gruttola V Gebski V Machin D First-Event or Marginal Estimation of Cause-Specific Hazards for Analysing Correlated Multivariate Failure-Time Data? Stat Med 2008 27 922 936 10.1002/sim.2944 17551931

[B21] de Bock GH Putter H Bonnema J van der Hage JA Bartelink H van de Velde CJ The Impact of Loco-Regional Recurrences on Metastatic Progression in Early-Stage Breast Cancer: A Multistate Model Breast Cancer Res Treat 2009 117 401 408 10.1007/s10549-008-0300-2 19148746

[B22] Rondeau V Mathoulin-Pelissier S Jacqmin-Gadda H Brouste V Soubeyran P Joint Frailty Models for Recurring Events and Death Using Maximum Penalized Likelihood Estimation: Application on Cancer Events Biostatistics 2007 8 708 721 10.1093/biostatistics/kxl043 17267392

[B23] Rondeau V Gonzalez JR Frailtypack: A Computer Program for the Analysis of Correlated Failure Time Data Using Penalized Likelihood Estimation Comput Methods Programs Biomed 2005 80 154 164 10.1016/j.cmpb.2005.06.010 16144730

[B24] Liu L Huang X The Use of Gaussian Quadrature for Estimation in Frailty Proportional Hazards Models Stat Med 2008 27 2665 2683 10.1002/sim.3077 17910008 PMC7364854

[B25] Cheung YB Gao F Khoo KS Age at Diagnosis and the Choice of Survival Analysis Methods in Cancer Epidemiology J Clin Epidemiol 2003 56 38 43 10.1016/S0895-4356(02)00536-X 12589868

[B26] Vaittinen P Hemminki K Risk Factors and Age-Incidence Relationships for Contralateral Breast Cancer Int J Cancer 2000 88 998 1002 10.1002/1097-0215(20001215)88:6<998::AID-IJC25>3.0.CO;2-0 11093827

[B27] Chen Y Thompson W Semenciw R Mao Y Epidemiology of Contralateral Breast Cancer Cancer Epidemiol Biomarkers Prev 1999 8 855 861 10548312

[B28] Gajalakshmi CK Shanta V Hakama M Risk Factors for Contralateral Breast Cancer in Chennai (Madras), India Int J Epidemiol 1998 27 743 750 10.1093/ije/27.5.743 9839728

[B29] Brinkmann D Ryan A Ayhan A McCluggage WG Feakins R Santibanez-Koref MF Mein CA Gayther SA Jacobs IJ A Molecular Genetic and Statistical Approach for the Diagnosis of Dual-Site Cancers J Natl Cancer Inst 2004 96 1441 1446 10.1093/jnci/djh272 15467033

[B30] Clarke M Collins R Darby S Davies C Elphinstone P Evans E Godwin J Gray R Hicks C James S MacKinnon E McGale P McHugh T Peto R Taylor C Wang Y Effects of Radiotherapy and of Differences in the Extent of Surgery for Early Breast Cancer on Local Recurrence and 15-Year Survival: An Overview of the Randomised Trials Lancet 2005 366 2087 2106 16360786 10.1016/S0140-6736(05)67887-7

[B31] Duchateau L Janssen P Kessic I Fortpied C Evolution of Recurrent Asthma Event Rate over Time in Frailty Models Journal of the Royal Statistical Society: Series C (Applied Statistics) 2003 52 355 363 10.1111/1467-9876.00409

[B32] Andersen P Gill R Cox's Regression Model for Counting Processes: A Large Sample Study Annals of Statistics 1982 10 1100 1120 10.1214/aos/1176345976

